# Signal detection in animal psychoacoustics: Analysis and simulation of sensory and decision-related influences

**DOI:** 10.1016/j.neuroscience.2012.06.001

**Published:** 2012-09-18

**Authors:** A. Alves-Pinto, J. Sollini, C.J. Sumner

**Affiliations:** MRC Institute of Hearing Research, Science Road, University Park, Nottingham, NG7 2RD, United Kingdom

**Keywords:** CR, correct rejection, dB, decibel, H, hit, ROC, receiver-operating characteristic, SDT, signal detection theory, SNR, signal-to-noise ratio, signal detection theory, ferret, behavioural detection, sensory, non-sensory

## Abstract

Signal detection theory (SDT) provides a framework for interpreting psychophysical experiments, separating the putative internal sensory representation and the decision process. SDT was used to analyse ferret behavioural responses in a (yes–no) tone-in-noise detection task. Instead of measuring the receiver-operating characteristic (ROC), we tested SDT by comparing responses collected using two common psychophysical data collection methods. These (Constant Stimuli, Limits) differ in the set of signal levels presented within and across behavioural sessions. The results support the use of SDT as a method of analysis: SDT sensory component was unchanged between the two methods, even though decisions depended on the stimuli presented within a behavioural session. Decision criterion varied trial-by-trial: a ‘yes’ response was more likely after a correct rejection trial than a hit trial. Simulation using an SDT model with several decision components reproduced the experimental observations accurately, leaving only ∼10% of the variance unaccounted for. The model also showed that trial-by-trial dependencies were unlikely to influence measured psychometric functions or thresholds. An additional model component suggested that inattention did not contribute substantially. Further analysis showed that ferrets were changing their decision criteria, almost optimally, to maximise the reward obtained in a session. The data suggest trial-by-trial reward-driven optimization of the decision process. Understanding the factors determining behavioural responses is important for correlating neural activity and behaviour. SDT provides a good account of animal psychoacoustics, and can be validated using standard psychophysical methods and computer simulations, without recourse to ROC measurements.

## Highlights

► Signal detection theory was applied to ferrets’ behaviour-detecting tones in noise. ► Sensory acuity was stable but the decisions depended on the set of stimuli. ► Decisions also depended on responses and rewards in previous trials. ► Computer simulations accounted for the data quantitatively. ► Behaviour reflects a stable sensory representation with a dynamic decision process.

## Introduction

Signal detection theory (SDT) is a quantitative framework to analyse subjects’ responses in psychophysical tasks (Green and Swets, 1966). It assumes that a subject’s ability to distinguish between different stimuli is limited by the variability of an (unspecified) internal sensory representation, and that behavioural responses are based on a comparison between the internal representation and a criterion value. Using certain assumptions sensory acuity (*d*′) can be derived, filtering out the influence of the cognitive decision process. Those assumptions underlying SDT have been tested in a wide range of psychophysical (and memory) experiments, particularly for human subjects, and generally hold (e.g. classically in hearing, Tanner et al., 1956). Nevertheless, there are discrepancies. Measured *d*′ can depend on the number of intervals and references in a forced choice task (Creelman and Macmillan, 1979) or differ between detecting change and discriminating the direction of change (Semal and Demany, 2006), and the appropriate sensory model is still debated (Kaernbach, 1991; Pastore et al., 2003; Micheyl et al., 2008).

SDT can also be applied to neural sensory responses, allowing quantitative comparisons between neural and psychophysical data (e.g. Britten et al., 1992; Parker and Newsome, 1998; Shackleton et al., 2003; Alves-Pinto et al., 2010). SDT-related models have been extended to coding in spike timing (Bizley et al., 2009; Malone et al., 2010) and to large neuronal populations (Pressnitzer et al., 2008; Alves-Pinto et al., 2010; Bizley et al., 2010). It is clear that neuronal responses reflect both sensory acuity and behaviour (e.g. Selezneva et al., 2006; Lee and Seo, 2007; Atiani et al., 2009; Zhou et al., 2010; Brosch et al., 2011).

Relating sensory neuronal and behavioural responses in the same species requires a thorough understanding of psychophysics in non-human animals (Stebbins, 1970; Dooling and Hulse, 1989). SDT is often used for analysis of behaviour (Marston, 1996; Alsop, 1998). However, although comparisons with physiology depend upon the SDT model, validation is attempted infrequently (Hack, 1963; Blough, 1967; Clopton, 1972; Marston, 1996; Talwar and Gerstein, 1999). The degree to which (and when) SDT is a reasonable model of psychophysical measurements in animals, is far from clear. Different methods yield systematic differences (Burdick, 1979; Klink et al., 2006), and SDT-derived measures of sensory acuity are subject to cognitive factors (Alsop, 1998).

The present study applied SDT techniques to analyse behavioural responses of ferrets in an auditory signal-in-noise detection task. Conventionally, the assumptions underlying SDT are tested by measuring the receiver-operating characteristic (ROC). This is done by attempting to manipulate the decision process and the discriminability of the stimulus independently, which is a lengthy process. It also requires SDT to hold under conditions of extreme response criterion not encountered in most psychophysical testing. Here, instead, we compare SDT analyses in two different methods of varying stimulus parameters, commonly used to measure psychophysical thresholds: the Method of Limits and the Method of Constant Stimuli. We show, with the aid of computational simulations, that SDT measures of sensitivity in the data are independent of stimulus presentation method. However, the decisions themselves are dependent upon both the set of stimuli presented in a session and on immediately preceding trials. Simulations, based on a SDT model of perception which accounts for several influences on decision criterion, accurately model these data.

## Experimental procedures

### Behavioural experiments

#### Subjects

Five adult pigmented ferrets (*Mustela putorius*), three females and two males, were trained and tested in a positive reinforcement behavioural procedure. Each underwent two behavioural sessions (20–40 min each) every day, during which they received most of their water intake. At the end of each day they were given ground ferret food mixed with additional water and a supplement (Cimicat, Petlife International Ltd., UK). One block of sessions typically lasted 11 days. After this water was available *ad libitum* for at least 3 days. All ferrets were weighed daily and their health monitored continuously. Behaviour was stopped if animals’ weight dropped 20% below their pre-regulation weight or if there were any other health concerns. Animals were housed individually with environmental enrichment in their cages and received daily social activity with other ferrets. All procedures were carried out under licence from the UK Home Office.

#### Behavioural apparatus

**Fig. 1:**
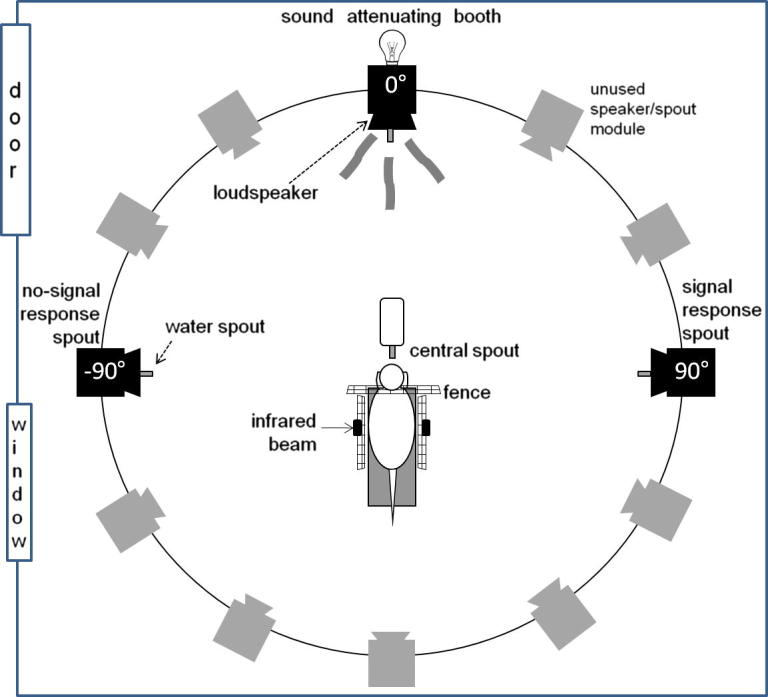
The apparatus used in the behavioural experiments. Black speaker/spout modules indicate those used for stimulus presentation (0°; including LED) and response/reward locations for no-signal (‘no’; −90°) and signal (‘yes’; 90°) trials. Grey modules were not used in this task. A continuous broadband noise and the target stimulus were played from the loudspeaker positioned at 0°. The ferret triggered a new trial by approaching the centre platform and licking the centre spout. When the target tone was presented (‘signal-trial’), the ferret could receive water reward at the 90° spout; if no tone was presented (‘no-signal trial’) then a reward was obtainable at −90°. The LED (indicated by the bulb) at 0° was illuminated on every trial.

Experiments were conducted in a circular arena (1.5 m diameter) inside a double walled, sound-attenuating room (IAC-1204, UK; Fig. 1). The inner walls were lined with mineral wool, except for a double-glass window. The arena floor was polyvinyl chloride (PVC) and the sides and top were made with wire mesh. The mesh perimeter was surrounded by acoustically transparent net fabric that concealed 12 custom-made modules (N.B. some of these modules were present for other studies; Irving et al., 2011). Each module contained a loudspeaker (Visatron FX10) and a solenoid-operated water spout, which contained an infrared lick detector. In the centre of the arena there was a platform with a water spout. The central spout registered a lick when the ferret touched the spout and the body simultaneously broke an infra-red beam. The beam ensured the ferret was correctly positioned on the platform. Additionally, the ferret put their head through a hole in a metal fence to lick the central spout, ensuring a consistent head position.

Stimulus presentation and response acquisition were done via the modules surrounding the arena, and were controlled by a MOTU 24 I/O system (Mark of the Unicorn, Cambridge, MA, USA) that was driven by a computer outside the booth using custom-made software. A custom-made USB system controlled the amount of water delivered (∼300 μL for each correct response).

#### Stimuli

The masker was a 48 kHz-wide noise played continuously, by looping a 30 s long frozen noise. The target used for testing was a 10 kHz pure tone, 500 ms long including 20 ms rise/fall ramps. The sampling frequency was always 96 kHz. The sound pressure level (RMS) was measured with a ½ in. B&K 4165 condenser microphone, pointing upwards and placed at a position where the ferret’s head was when a trial was triggered. The sound pressure level of the background noise was 35 or 55 dB SPL for ferret F1 and 48 dB SPL for ferrets F2–F5.

#### Behavioural task

Ferrets were trained to perform a ‘yes/no’ detection task (Neff et al., 1975; Kelly et al., 1986; Hine et al., 1994), initiating trials by licking the central spout. On half the trials a 10-kHz tone was presented (‘signal trials’) simultaneously with a visual timing cue (the illumination of an LED in the 0° module). In the rest of trials the LED was illuminated but no sound presented (‘no-signal trials’). Signal trials were rewarded when responses were made at the spout located at 90° (‘yes’ response), whilst no-signal trials were rewarded at the −90° spout (‘no’ response). Incorrect responses were not rewarded, and the subsequently triggered trial was identical to the previous. These ‘correction trials’ continued until a correct response was given.

Training began with the ferrets learning to lick and obtain reward from each of the three water spouts in silence, and gradually to lick the centre spout whilst lined up facing forward on the platform. Next, the detection task was introduced. Licking the centre spout initiated a trial as described above, and this was rewarded on every trial whilst only correct responses were rewarded at the answer spouts. The level of the tone was fixed at 65 or 72 dB SPL. In early training the tone continued until the ferret responded. Following 2–3 consecutive sessions of high performance (>90% correct), the tone duration and/or the amount of water given at the centre were reduced. This continued progressively until the tone was 500 ms long and only two drops of water were given at the centre, randomly on one in eight trials. The continuous background noise was then introduced directly at the level used during testing (48 or 55 dB SPL). Complete training of a ferret usually lasted 2–3 months.

#### Psychophysical procedure and estimation of detection threshold

The testing procedure differed from training in that the level of the target tone was varied to yield a psychometric function and a detection threshold. Performance was measured with three different methods, differing in the way the level of the target tone was varied: (1) Method of Limits, (2) Method of Constant Stimuli, and (3) Adaptive Tracking.

##### Method of limits

The level of the tone was fixed within a session and decreased systematically across a contiguous block of sessions until performance was below threshold (Fechner, 1948; Kelly et al., 1986; Hine et al., 1994; Lam et al., 1996). The rates of hits (‘yes’ on a signal trial) and false-alarms (‘yes’ on a no-signal trial) were calculated for the trials in each session, ignoring correction trials and any trials where water was delivered at the centre spout. *d*′ values were calculated (Green and Swets, 1966) from these, and the corresponding percentage correct values for an unbiased observer, *P*(c)_max_ (MacMillan and Creelman, 2005).

At the start of a contiguous block of sessions, the tone was set at a high level. Following 2–3 sessions of *P*(c)_max_ > 90%, the level of the tone was reduced by 10 dB. The level of the tone was then reduced by 10 dB each session until *P*(c)_max_ dropped below 85%. Subsequently the tone level was decreased in 5 dB steps every other session until *P*(c)_max_ fell below 71%, with a third session at a given level if *P*(c)_max_ varied by more than 10%. On average, the proportion of correction trials per behavioural session increased (from 6% to 27%) as the signal-to-noise ratio (SNR) decreased (24 to −15 dB). The average number of rewarded trials in a session remained approximately the same at all SNRs. The total number of trials increased with decreasing SNR as the proportion of correct, rewarded, trials decreased.

For subsequent analyses, *P*(c)_max_ values at a given level were derived from the overall hits and false alarm rates calculated from two or more consecutive sessions, with a minimum of 50 signal trials and 50 no-signal trials. At least two blocks of sessions with the Method of Limits were collected for every ferret, interleaved with blocks using other methods. Psychometric functions of *P*(c)_max_ as a function of tone level were fitted with logistic functions, from which detection thresholds were derived for a *P*(c)_max_ of 71%.

##### Method of Constant Stimuli

In a block of sessions using the Method of Constant Stimuli, a preset group of 4–6 tone levels were tested in each session. The set of levels was set, with some piloting, so that at least one was easily detectable and one was below threshold. A proportion of easily detectable signals served to monitor suprathreshold performance and help maintain the correct stimulus–reward relationship. Psychometric functions were derived from (three or more) sessions over which there were a minimum of 50 signal trials per each of the levels tested. For each signal level a corresponding false alarm rate was calculated from a randomly selected sample (matched to the number signal trials) of the available no-signal trials in those sessions. This way, both hit and false alarm rates were calculated from a similar number of trials, as occurs also in the Method of Limits. On average, 21% of trials were correction trials.

##### Adaptive Tracking procedure

In a block of sessions using the Adaptive Tracking (up-down) procedure, the level of the signal was varied during a behavioural session according to the subject’s response outcome: signal level was reduced (down) after a correct response, and increased (up) after an incorrect response (Levitt, 1971). In the beginning of the session the level of the tone was kept high and was reduced (6 dB) only after five consecutive correct responses to either signal or no-signal trials (1-up/5-down). Afterwards, the level of the tone was increased (4 dB) after an incorrect response and decreased (4 dB) after two consecutive correct responses (1-up/2-down rule**)**. This continued for 4–6 reversals and afterwards the level of the tone was changed by 2 dB, using the same 1-up/2-down rule. Thresholds were derived by averaging the tone levels at reversal points in the last part of the adaptive track.

#### Analysis of trial-by-trial dependency of decisions

We computed the probability of a ‘yes’ response on the current trial *i* for different outcomes in the previous trial *i* − 1: hit, correct rejection (CR; ‘no’ to a no-signal trial), miss (‘no’ on a signal trial) or false-alarm. This was done separately for signal and no-signal trials, and for each signal level. Probabilities were first calculated for individual sessions and then averaged across sessions.

For example, the probability of a ‘yes’ response on trials where a signal was presented at 0 dB SNR was split into three different probabilities: (1) after a hit (*P*[yes*_i_*_,signal_|Hit*_i_*_−1_]), (2) after a correct rejection (*P*[yes*_i_*_,signal_|CR*_i_*_−1_]) and (3) after a miss (*P*[yes*_i_*_,signal_|Miss*_i_*_−1_]). After a (unrewarded) miss would follow a correction trial, so an opposite response (a rewarded ‘yes’) would be unsurprising. However, a significant difference in the probability when the previous trial was a hit versus a correct rejection indicated the response depended on the previous trial, despite signal and no-signal trials being equally likely. Note that owing to correction trials, a signal trial cannot follow a false-alarm, so this probability does not exist. A similar analysis can be done when the current trials are no-signal trials, in which case the outcome in the previous trial is limited to hits, correct rejections and false-alarms. This analysis was also extended to the dependency on the outcome two trials back (*i*−2).

### Computational model based on signal detection theory

A SDT model was used to simulate the ferrets’ behaviour. Each simulated trial generates a sensory value, the ‘internal representation variable’. For signal trials this was the dB SPL of the signal plus some variability, or internal-noise. For no-signal trials it was internal noise plus a parameter (‘reference level’) determining the signal level which was indistinguishable from no-signal trials. Variability was modelled as a Gaussian-distributed random number, with a fixed standard deviation in dB (‘internal s.d.’) for all trials.

The decision in a given trial was based on the comparison between the internal representation variable and a decision criterion (in dB SPL). The decision criterion was a combination of an *optimal* criterion, and a systematic shift away from this. The optimal point was not a parameter but was calculated as the point between the internal no-signal distribution and signal distributions at which the probabilities of a false alarm and a miss are equal. For the Method of Limits, the signal distribution was that of the signal level used in that session and the optimal point is just midway between the two distributions. For the Method of Constant Stimuli, signal distribution was taken as the average of the distributions associated with the different tone levels tested in a session (Gorea and Sagi, 2000) and the optimal criterion is below the intersection point.

Shifts away from the optimal decision criterion were modelled as a fixed shift in dB. This was fixed for a given block of (∼20) behavioural sessions (‘criterion bias’). All the other parameters were fixed across all simulations for a given ferret and method. The dependency on the previous trial (Figs. 5 and 6) was modelled by shifting the criterion by a fixed amount (in dB, the ‘trial shift*_i_*_−1_’) on each trial, added if the answer on the previous trial was ‘yes’ and subtracted if it was a ‘no’. A second ‘trial shift*_i_*_−2_’ parameter accounted for the dependency on the trial before that. We also included an attentional factor (‘guess-rate’): a propensity to guess on a proportion of trials, with a 50% probability of choosing either answer regardless of the sensory information in that trial (Wightman and Allen, 1992). Inattention tends to bring down the top end of the psychometric function, reducing the maximum performance for supra-threshold stimuli.

For each ferret and method, the parameters were adjusted to minimise the mean squared error for hit and false-alarm rates between the simulations and the data (see Table 1 for a description). The number of trials in each session approximated the average for each ferret, and errors were calculated from 100 simulated sessions for every set of stimulus conditions and model parameters. Fitting was done in several supervised stages, using both mapping of parameter ranges and automatic minimisation on subsets of parameters. Initially, the parameters ‘internal s.d.’, ‘reference level’ and ‘criterion bias’ were optimised whilst the remainder were fixed (trial shift*_i_*_−1_ = 2; trial shift*_i_*_−2_ = 1.5; guess rate = 1%). Subsequently the ‘guess-rate’ was optimised by simulating a pre-set range of values and finally trial dependencies (trial shift*_i_*_−1_ and trial shift*_i_*_−2_) were optimised in the same way (these parameters had little influence on hits and false alarms).

To assess the success of the model we correlated the *P*(c)_max_, hit rates and false-alarms from each behavioural block against the SNR-matched model output, averaged across many sessions (200 per SNR for the Method of Limits, 1000 for the Method of Constant Stimuli). Part of the variability in both the data and the simulations was a product of the number of trials and tone levels in a behavioural session. Therefore we also correlated the model against itself. We repeatedly (100 times) drew one block of sessions from the total pool of simulations and correlated it with the mean across the remainder. This gave an upper limit on the possible correlation given the experimental protocol. Correlations of false-alarm rate were not considered for the Method of Constant Stimuli as these would be close to zero and not meaningful, since no-signal trials are not associated with any SNR in this method. 95% confidence intervals for *P*(c)_max_, hit rates and false-alarms were also calculated from these simulations.

## Results

### Methods of Limits and Constant Stimuli yield similar psychometric functions and detection thresholds

**Fig. 2:**
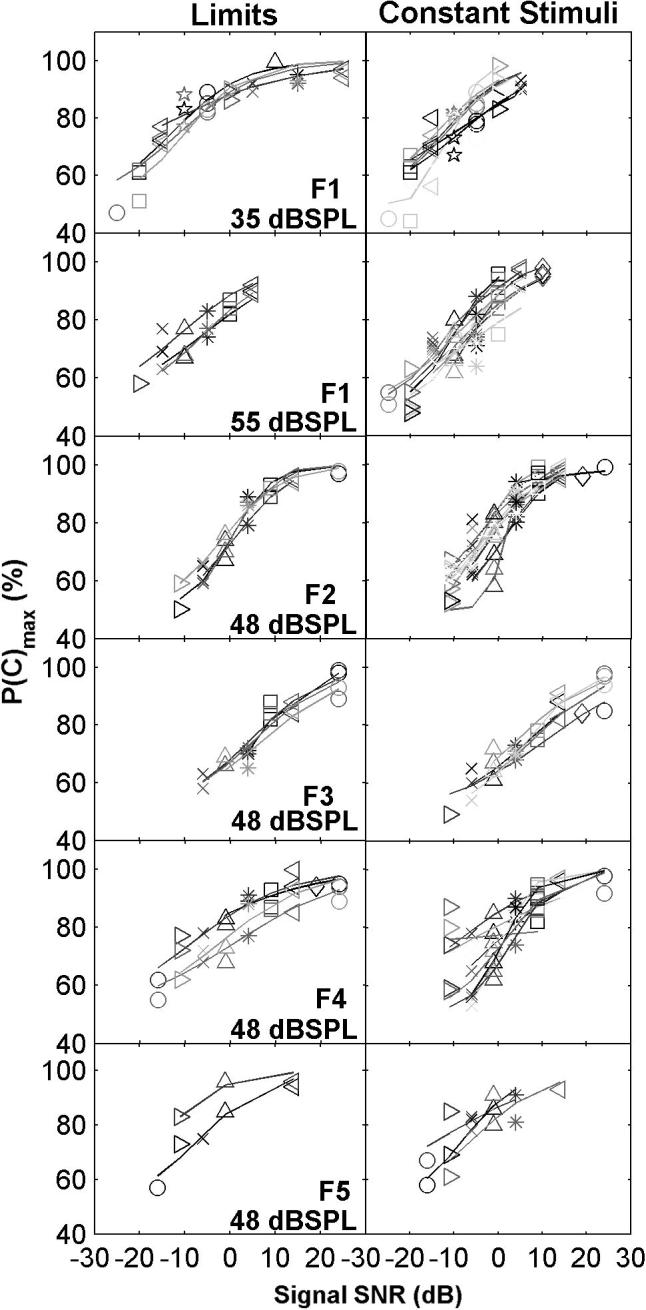
Psychometric functions collected for individual ferrets with the Methods of Limits (left column) and Constant Stimuli (right column). Each row illustrates results for a different subject and/or masker level. Different symbols indicate *P*(c)_max_ values at different SNRs (dB). Different shades of grey distinguish separate blocks of behavioural sessions. Lines are logistic fits to experimental values.

**Fig. 3:**
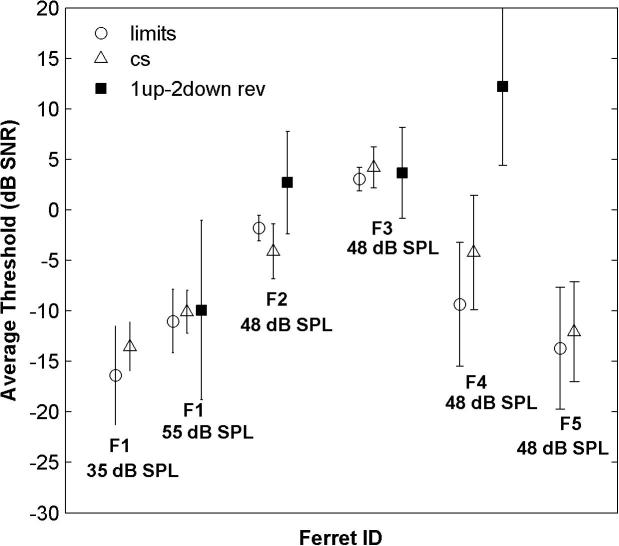
Average signal detection thresholds (dB SNR) obtained with the Method of Limits (circles), Constant Stimuli (triangles) and Adaptive Tracking (squares). Average thresholds and standard deviations were calculated across the different blocks of behavioural sessions (i.e. each psychometric function).

SDT posits an underlying and unchanging sensory representation. Consistent with this, psychometric functions, expressed as the criterion-free measure *P*(c)_max_, were not significantly different whether obtained with the Methods of Limits or Constant Stimuli (Fig. 2; *p* > 0.05; one-way MANOVA applied to *P*(c)_max_ at different SNRs), except for one ferret (F3). However, there were differences between individuals (*p *< 0.05; ANOVA on ferret × method × SNR). The similarity in the shape of the psychometric functions obtained with the Methods of Constant Stimuli and Limits was confirmed by the match of tone detection thresholds (Fig. 3). Thresholds differed between individuals, but did not differ between the two methods (*p* > 0.05; two-way ANOVA on ferret × method).

We also attempted to measure thresholds with an adaptive tracking procedure. However, similar to observations in mice (Klink et al., 2006), this yielded more variable threshold estimates than the other two methods, and in several cases higher thresholds (squares in Fig. 3). We did not therefore attempt to analyse the adaptive tracking data further.

### Hit and false-alarm rates for Methods of Limits and Constant Stimuli show different distribution patterns

In SDT, the optimal decision criterion will be different if signals are presented at only a single high suprathreshold level, or at a low level, or if the signal level varies from trial-to-trial. Human subjects can only maintain a single internal representation and one decision criterion in a given task (Gorea and Sagi, 2000). Thus, although the method used did not significantly affect criterion-free measures of performance, we expect it to affect hit and false-alarm rates.

**Fig. 4:**
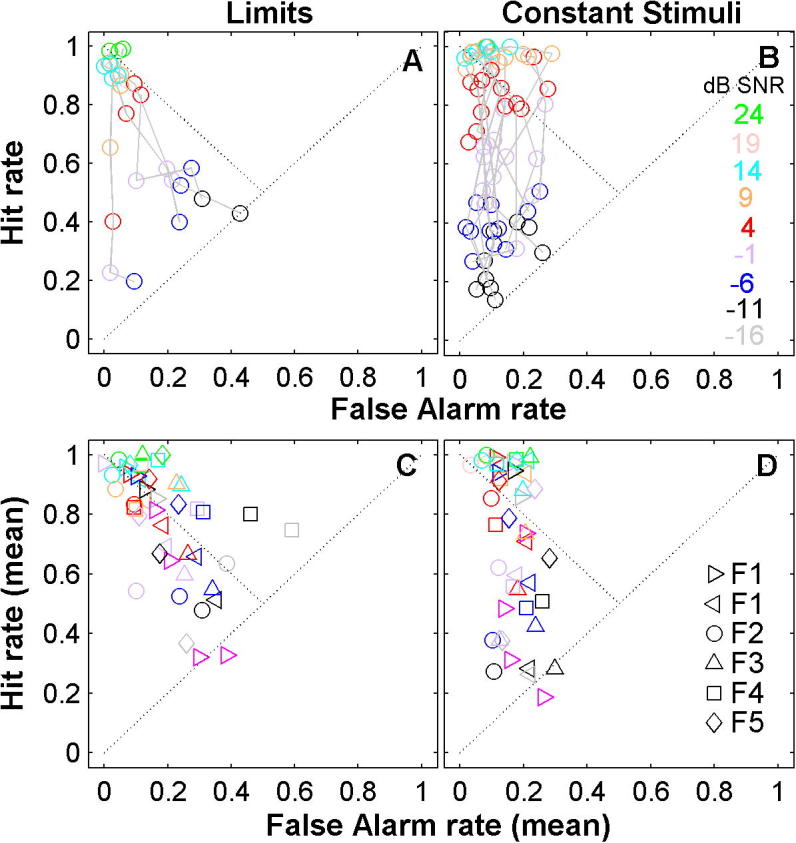
Hit rate versus false-alarm rate. Lines join data collected within a single block of sessions. Colour differentiates signal SNR (dB) as indicated in the inset. (A) Method of Limits for F2. (B) Method of Constant Stimuli for F2. Lower panels (C, D) show data for all animals. Symbols differentiate ferrets; colour differentiates tone level, as in (B). Each point corresponds to the *mean* hit/false-alarm rate, for a given ferret, averaged across all blocks of sessions. Dotted lines mark chance (hit rate = false-alarms) performance and unbiased (hit rate = 1-false-alarms) behaviour. (For interpretation of the references to colour in this figure legend, the reader is referred to the web version of this article.)

The distribution of hit rates against false-alarm rates is shown for ferret F2 for the Methods of Limits (Fig. 4A) and Constant Stimuli (Fig. 4B). In this ferret false-alarm rates were lower than hit rates for both methods. However, for the Method of Limits false-alarms increased with decreasing SNR, whilst for the Method of Constant Stimuli the false alarm rates remained virtually constant across signal level because, unlike the Method of Limits, no-signal trials are not associated with signal trials of a specific SNR. A one-way MANOVA of the hits and false-alarms confirmed there was a significant effect of data collection method (*p* < 0.05) for this ferret.

For all ferrets, individually, there were significant differences between the methods for both hits and false-alarms (*p* < 0.05, one-way MANOVA as above). Fig. 4C and D shows data averaged across blocks of sessions for each ferret. For the Method of Limits, all ferrets except F5, for which a small number of sessions were collected, had significantly higher false-alarm rates at low signal levels (one-way ANOVA for all ferrets separately, *p* < 0.05), indicating that they were shifting their decision criterion towards the optimal unbiased point. For the Method of Constant Stimuli, the average false-alarm rate was around 20% although false-alarm rates did differ across ferrets (two-way ANOVA, *p* < 0.05).

Taken together with the invariance of *P*(c)_max_, this suggests that SDT provides a clear interpretation of the animals’ sensitivity in this detection task: the underlying sensory representation is relatively constant within individual ferrets, and does not depend on the data collection method. However, the decision strategy differs within animals depending on the data collection method (i.e. the set of stimuli presented).

### Behavioural responses show sequential dependencies

Decision processes that determine psychophysical responses are not necessarily static. In particular, they can depend on the outcome on previous trials (e.g. Ward and Lockhead, 1970; Jesteadt et al., 1977; Treisman and Williams, 1984; Petzold and Haubensak, 2001). This can be interpreted in SDT as a change in the decision criterion from trial-to-trial (Treisman and Williams, 1984). Such effects have been reported rarely in animals during psychoacoustic testing (Talwar and Gerstein, 1999), but have been more commonly reported in tasks that set out to manipulate trial-by-trial reward expectation (e.g. Blough and Blough, 1985; Barraclough et al., 2004; Lau and Glimcher, 2005; Brosch et al., 2011).

**Fig. 5:**
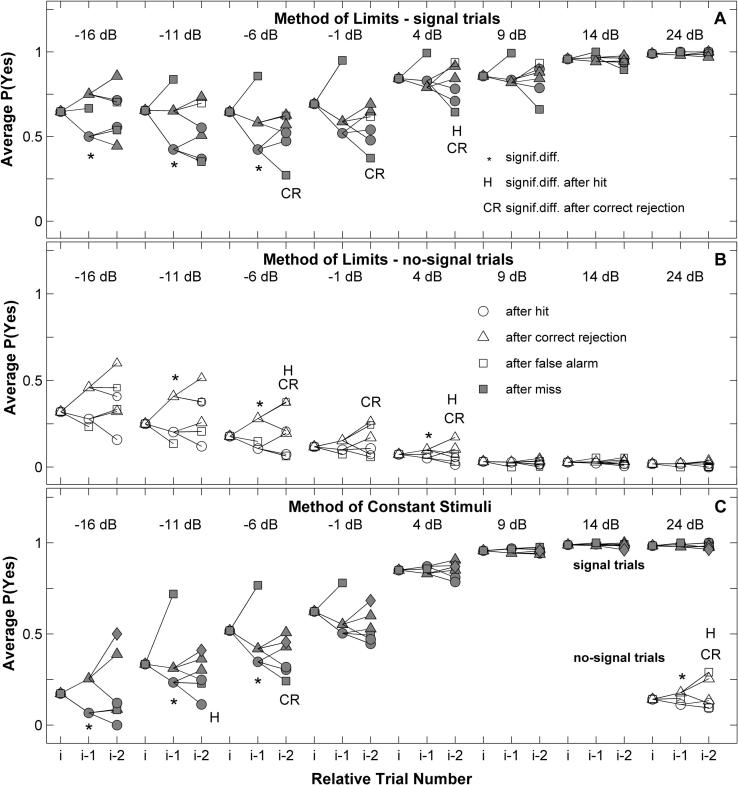
The dependency of responses on the outcome of previous trials for ferret F2. (A) Signal-trials for the Method of Limits. Each tree shows a different SNR. The leftmost symbol in each tree indicates the overall probability of a ‘yes’ response, *P*(yes), averaged across sessions. The middle points represent *P*(yes), averaged across sessions, after a hit (●, *P*[yes*_i_*| Hit*_i_*_−1_]), a correct rejection (▴, *P*[yes*_i_*|CR_i−1_]), and a miss (■, *P*[yes*_i_*| Miss*_i_*_−1_]). The rightmost points in each tree show the dependency on the last but one trial. For example, the filled triangle (▴) right and uppermost in a tree give the average *P*(yes) given the previous trial was a CR and the previous to that one a CR: *P*[yes*_i_*_,signal_|CR*_i_*_−1_|CR*_i_*_−2_]. Asterisks indicate significant dependencies on the previous trial (*i*−1) between hits and correct-rejections; ‘H’ and ‘CR’ indicate significant dependency on two trials previous (*i*−2) when the previous trial (*i*−1) were hits and correct rejections. (B) No-signal trials for the Method of Limits. Symbols as (A) except for false-alarms (□; *P*[yes*_i_*|FA*_i_*_−1_]). (C) Method of Constant Stimuli.

Fig. 5 shows the trial dependencies observed for ferret F2, as a separate tree for signal and no-signal trials at each SNR. The overall probability of a ‘yes’ response (leftmost symbols in each tree) is split into separate probabilities dependent on the previous trial (middle symbols in each tree), and the one before that (rightmost symbols). On signal trials the ferret was more likely to give a ‘yes’ response if the previous trial was a correct rejection (▴) than if the previous trial was a hit (●; ∗ indicates a significant difference after Holm–Bonferroni corrected Chi-squared test). This pattern was reversed for no-signal trials. Effectively, it showed a tendency to respond at the spout opposite to that at which it had just received a reward from, despite the a priori probability of a reward being equal. This dependency was stronger at lower SNRs (*F* = 13.3 after linear regression model). We were less interested in trials following incorrect answers, since the same trial was presented again (a correction trial), so the correct response was simply the opposite of the previous response. Consistent with this, the highest proportion of ‘yes’ responses and ‘no’ responses usually occurred after a miss (■) and a false-alarm (□) respectively. However, below −1 dB SNR for signal and no-signal trials these proportions were significantly different from 1 or 0 (Kolmogorov–Smirnov test on the mean proportion of correct responses calculated for each behavioural session at which a given SNR was tested, *p* < 0.05), indicating that this ferret did not always respond correctly on correction trials.

Decisions also depended on the previous trial for data collected with the Method of Constant Stimuli (Fig. 5, lower panel). The probability of a ‘yes’ response after a correct rejection was again larger than after a hit from −16 to −6 dB SNRs (Holm–Bonferroni corrected Chi-squared test). For no-signal trials, which were presented together with signal trials of different tone levels within the same session, it was not possible to separate out the effect of signal level. Nevertheless, the overall dependency was significant (Chi-squared test, *p *< 0.05) for no-signal trials (Fig. 5, lower panel, right corner).

The dependency on the outcome two trials prior (Fig. 5) showed a very similar pattern of results, for both methods of data collection. For signal trials, a ‘yes’ was more likely after a correct rejection in trial *i* − 2 than after a hit, whether there was a hit (H indicates significance in Fig. 5) or a correct rejection in trial *i* − 1 (CR indicates significant difference). Dependencies of trials further back were not significant due to the small number of trials for each combination of dependencies.

**Fig. 6:**
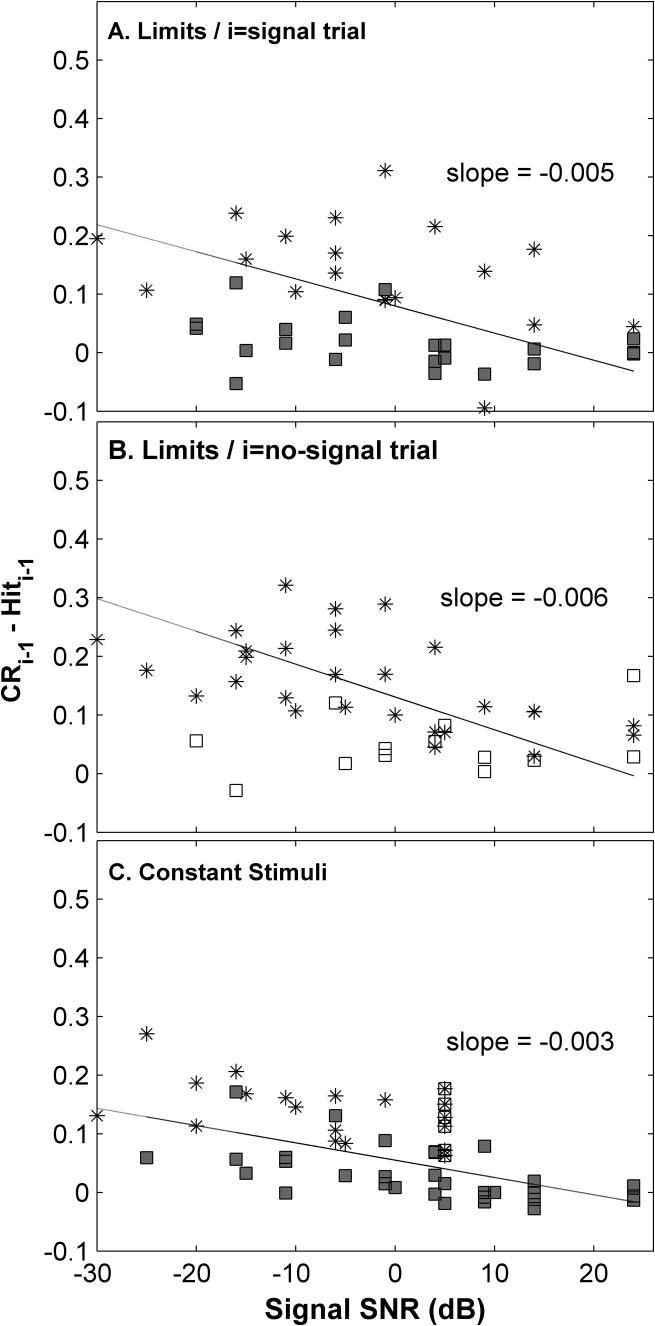
Difference between the probabilities of a ‘yes’ response after a correct rejection (CR) relative to that after a hit, as a function of signal-to-noise ratio, across ferrets. (A) Signal trials for the Method of Limits. (B) No-signal trials for the Method of Limits. (C) Method of Constant Stimuli (■, signal trials; □, no-signal trials). Asterisks indicate significant differences (Holm–Bonferroni corrected Chi-squared test, *p* < 0.05). Also indicated is the slope of the linear regression to all points in the panel.

Significant trial-to-trial dependencies were observed for all subjects tested (Fig. 6; * indicates corrected Chi-squared test) and were generally more frequent and larger at lower SNRs. However, this effect of level was not significant (Fig. 6; e.g. one-way repeated-measures ANOVA, *F*(1, 1) = 13.2, *p* = 0.18, for the Method of Constant Stimuli).

These dependencies can be interpreted within SDT as trial-by-trial shifts in decision criterion: the criterion is lowered following a correct rejection and raised following a hit. The stronger influence of previous trials in the current response at lower levels is expected, since the internal representations of signal and no-signal trials overlap more at low levels. The level of the signal also affects dependencies in humans (Speeth and Mathews, 1961). We did not set out to encourage them but these dependencies could arise as a consequence of the correction trials by encouraging ferrrets to go to the opposite spout on the next trial. Such trials are used widely during behavioural testing in animals (e.g. Blough and Blough, 1977; Kelly et al., 1986; Britten et al., 1992; Maier et al., 2010) to control for response biases and to ‘instruct’ errors. We tested the influence of correction trials on these dependencies by measuring with ferret F2 a full psychometric function with the Method of Limits *without* employing correction trials. Data (not shown here) showed similar trial-to-trial dependencies. Although this result does not rule out a role for correction trials in the development of these dependencies, it suggests that their use in individual sessions is not instrumental in producing them.

### A SDT model of behavioural responses and psychometric functions

A computational model based on SDT was used to simulate behavioural responses of each ferret. The goal was to see if we could account quantitatively for the patterns of observed hits and false-alarms, the measured psychometric functions and the differences between the two methods of data collection. Good fits to the data would constitute further evidence that the underlying SDT model was sufficient in the context of practical psychophysical measurements. It also allowed us to assess how optimal decision criteria were in both methods. Further, we wanted to estimate the different sources of variability required to account for the data. Variability will arise from noisy sensory representations, but may also be introduced by the trial-by-trial dependencies, longer term shifts in decision criterion, inattention, and the number of behavioural trials.

Each simulated trial elicited an internal representation on a dB scale, with a degree of internal variability (‘internal s.d.’ parameter). The optimal decision criterion was assumed to be such that the probability of a false alarm equals the probability of a miss (in a given behavioural session). Decision criterion could shift systematically away from the optimal (‘criterion bias’), as well as having a dependency on the previous two trials (‘trial shift’ parameters). The model also ‘guessed’ responses randomly on a proportion of trials (‘guess rate’ parameter), to account for possible inattention.

**Fig. 7:**
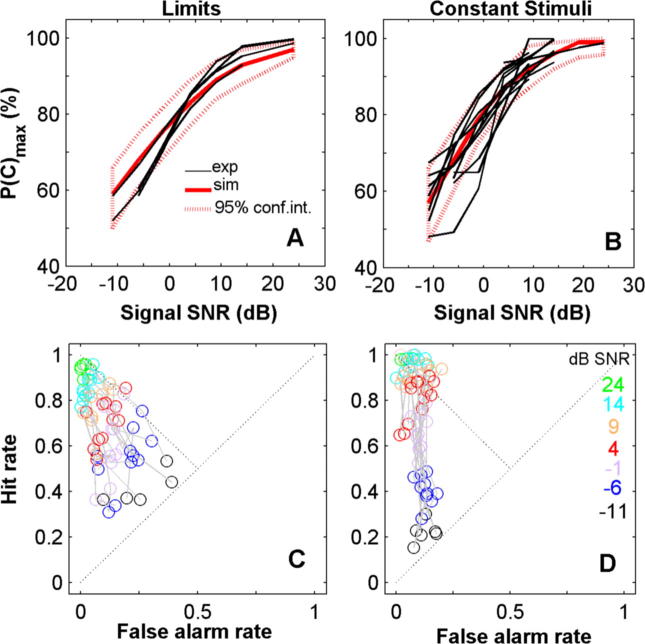
Comparison between experimental data and simulations for ferret F2. (A, B) Average simulated psychometric function (red line) and experimental functions (thin black lines) for the Method of Limits (A) and Method of Constant Stimuli (B). Dashed red lines show 95% confidence regions for the simulations. (C, D) Simulated hit and false-alarm rates. Each line illustrates results for a different block of sessions and colours indicate different signal SNRs, as in Fig. 5, with the number of simulated trials matched to the data average. (For interpretation of the references to colour in this figure legend, the reader is referred to the web version of this article.)

The average simulated psychometric functions reproduced the main features of the experimental functions for both methods (shown for F2 in Fig. 7A and B) although, for example, some of the data points for F2 fell outside of the 95% confidence bounds of the model. The shape of the functions was largely determined by the variability in the internal representation (internal s.d.). Across all ferrets, the fitted model values associated with the sensory representation were very similar between methods (Table 1; difference between methods: internal s.d. 0.54 ± 1.7 dB; reference level 0.5 ± 3.01 dB), offering further evidence that sensory representations were stable between methods.

The simulations also captured the main features of the hit and false-alarm rates (Fig. 7C and D for ferret F2, see Table 1). To quantify the success of the models in accounting for the data (independently of the fitting process), each block of experimental sessions was correlated (for matched SNRs) against the corresponding mean simulated *P*(c)_max_, hit and false-alarm rate values (see Table 1, column ‘exp–sim’; see methods). A large degree of the variability in *P*(c)_max_ (mean 82% of the variance), hit rate (78%) and a lesser degree of false-alarms (57% of the variance) is accounted for by the fitted models. However, these are in fact only slightly worse (10% worse on average) than for correlations calculated for the model against itself (Table 1, column ‘sim–sim’), indicating that much of the remaining variability can be accounted for by the experimental protocol.

For both methods, the fitted parameters showed systematic shifts in criterion, smaller for the Method of Limits, towards a higher than optimal criterion (Table 1: mean ‘criterion-bias’ across blocks is positive in all cases). Nevertheless, this is much smaller than the width of the internal distribution, and suggests that ferrets adopted a near optimal strategy for a given ‘mean’ signal distribution.

Another aspect of behaviour made clear by the simulations is that the proportion of random guesses in ferrets’ responses was low (5% or less). This can also be seen in the data. For most ferrets *P*(c)_max_ values converge to near 100% at high signal levels (Fig. 2). Thus, there is little evidence that a lack of attention influenced ferrets’ responses, and this parameter contributed very little to the quality of the fits.

Two additional parameters, ‘trial shift*_i_*_−1_’ and ‘trial shift*_i_*_−2_’, determined the dependency on previous trials. Trial dependencies were fitted subsequent to the other parameters, and had very little effect on the fits to the hit and false-alarm rates. Although not shown, in the model the effect of signal level on the trial-to-trial dependencies emerges naturally, since the internal representations of high level signal-trials are well spaced from no-signal trials. The responses for hits and correct rejections are reproduced well by the model. The responses following misses and false-alarms, which are correction trials, are not. The responses on correction trials neither reflect the ferret realising that they had made a mistake (which would give ‘yes’ response probabilities of 0 or 1), nor are they consistent with the same criterion shift as is observed following a correct trial. Thus, trial-to-trial shifts in criterion were contingent on both the response and the reward. We did not attempt to simulate the effect of correction trials more accurately, which would have required additional parameters. Across ferrets, shifts were slightly larger for the Method of Constant Stimuli than for the Method of Limits (see Table 1; difference between methods: 0.4 ± 0.5 for trial shift*_i_*_−1_ and 0.5 ± 0.6 for trial shift*_i_*_−2_).

### The effect of trial-to-trial dependencies on the shape of the psychometric function

**Fig. 8:**
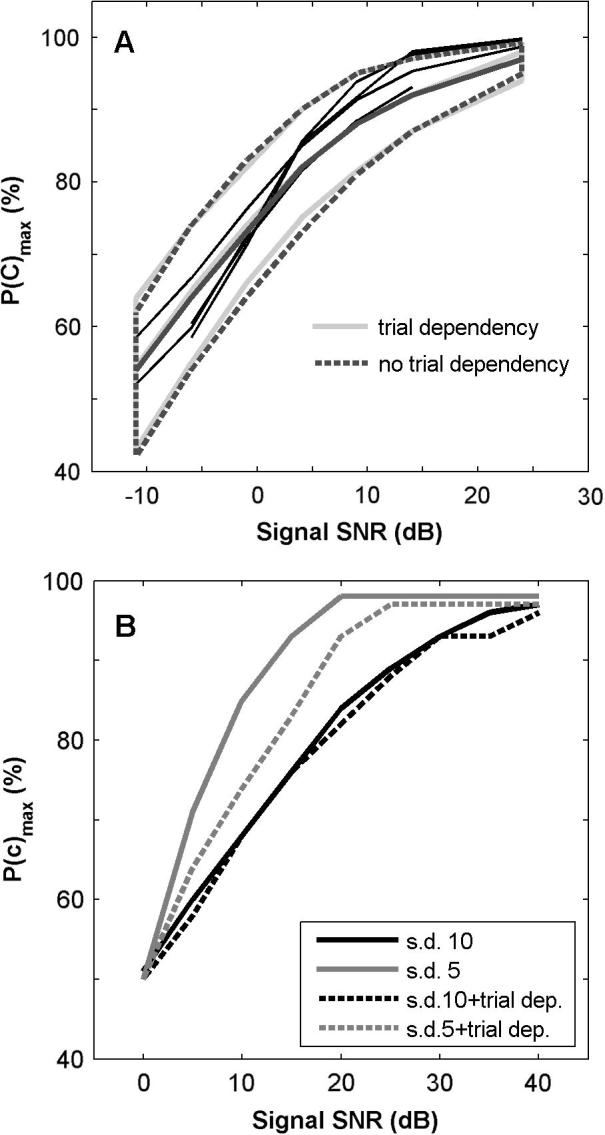
(A) Simulated (thick lines) and experimental (thin black lines) psychometric functions obtained and measured for ferret F2, for the Method of Limits. Thick central lines illustrate the median simulated psychometric functions and outer lines define 95% confidence intervals with (grey line) and without (dashed line) trial-to-trial dependencies in the simulations. (B) Effect of trial dependencies on example model psychometric functions for similar variability of internal representation to the data (internal s.d. = 10), and less (s.d. = 5). Simulated trial shift is 5 dB for the previous trial only.

The trial-to-trial dependencies could affect the shape of the psychometric function and therefore signal detection threshold. The likely influence on the data was evaluated using the model fitted to the behavioural responses of ferret F2 (Fig. 7), and comparing the psychometric functions simulated with and without (trial shift = 0) dependency effects. Median psychometric functions and 95% confidence intervals, calculated from 800 simulated behavioural sessions showed that the introduction of the dependencies produce only minimal changes in *P*(c)_max_ values at low signal levels (Fig. 8A shows simulations of the Method of Limits), and no change (46.3 versus 46.6 dB SPL) in average detection threshold. Hence, trial-to-trial dependencies did not affect average thresholds despite the changes produced in the distribution of criterion values. Neither did these parameters affect the quality of the fit to the hits and false-alarm rates.

Further exploration of model parameters (Fig. 8B) showed that psychometric functions become shallower, when trial-dependencies are large relative to the variability in the internal representations (‘internal s.d.’ parameter). Thus the effects of trial-dependencies on psychometric functions are very similar to variability in the internal representation, but the size of the measured trial-dependencies and the slopes of the psychometric functions suggest that this is not affecting either psychometric function shapes or thresholds.

### Reward maximisation as a mechanism for setting decision criterion

The model makes assumptions about where criterion should be set, but it does not explain how the ferrets might optimise their decision criteria. Here we attempt to relate shifts in overall decision criterion to the maximisation of reward.

**Fig. 9:**
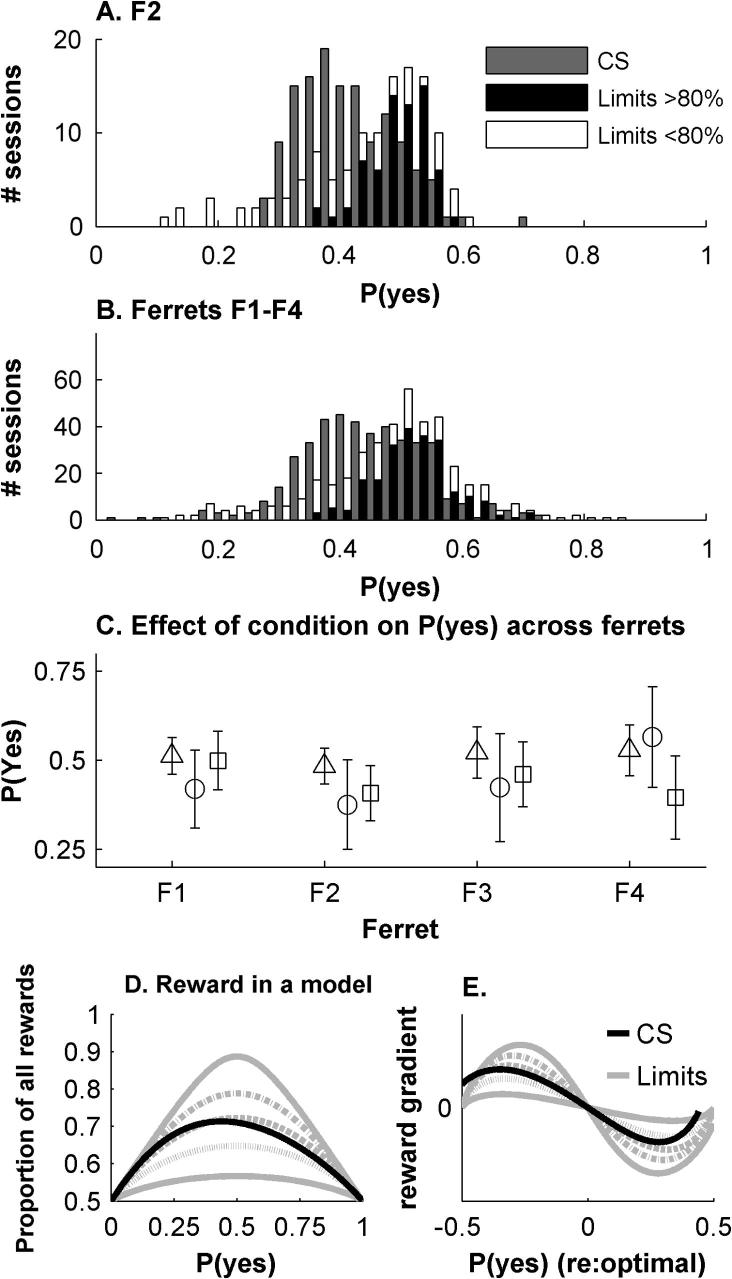
Proportions of ‘Yes’ responses and the relationship to rewards. (A) *P*(yes) for ferret F2, for Method of Limits (black: sessions >80% correct; white: sessions <80% correct) and the Method of Constant Stimuli (grey). (B) As (A) for ferrets F1–F4 together. (C) Mean and s.d. of *P*(yes) for each ferret, across Method of Limits (△ sessions >80% correct; O sessions <80% correct) and Method of Constant Stimuli (□). (D) Proportion of rewards versus *P*(yes) in an SDT model, for the Method of Limits at various SNRs (dB; grey lines – peak increases with SNR) and the Method of Constant Stimuli (black line) with the same set of SNRs. (E) Gradient of reward change versus *P*(yes) in the model (aligned for zero reward gradient). Model parameters in (D) and (E) are fitted to F1 for the Method of Limits.

Fig. 9 shows, for ferret F2 individually (panel A) and ferrets F1–F4 together (B, C), the proportion of ‘yes’ responses (excluding correction trials) across behavioural sessions, for each method. Method of Limits data are further split into sessions where performance was above (i.e. high SNRs) or below 80% correct (low SNRs). *P*(yes) differed between these three groups of conditions, as well as between ferrets (two-way ANOVA, *p* < .0001). For sessions using the Method of Limits where performance was high (black bars in Fig. 9A, B; triangles in C) *P*(yes) clusters around 50% correct, which would be optimal for an equal variance SDT model. However, in sessions where performance was lower (open bars in Fig. 9A, B; open circles in C) *P*(yes) was lower (*p* < .0001, *t*-test on F1–F4 together, Bonferroni corrected) and the distribution of responses more variable (*p* < .0001, variance test). For the Method of Constant Stimuli, *P*(yes) was again lower (*p* < .0001 for *t*-test) and more variable (*p* < .0001) than the high performance Method of Limits Sessions, though not as variable as the low performance Method of Limits sessions (*p* < .0001). These differences were largely evident in individual ferrets (though note F4).

Fig. 9D plots the proportion of rewards obtained (1 being 100% correct) for a deterministic version of the SDT model (i.e. signal and no-signal distributions bisected by a static decision criterion), as a function of *P*(yes). Each line was obtained by varying the decision criterion across the full range of both signal and no-signal distributions. The grey lines show that, for varying SNRs with the Method of Limits, rewards are always maximised when *P*(yes) = .5. Thus, the consistently low *P*(yes) seen in the more difficult Method of Limits sessions appear at odds with the model. The lower *P*(yes) of data collected using the Method of Constant Stimuli, on the other hand, is consistent with the model: reward is maximised by a slightly conservative criterion.

There is one possible explanation for the variable decision criterion setting in the data (Maddox, 2002). Fig. 9E shows, using the same model, the gradient of the reward function against *P*(yes). The benefits of shifting criterion naturally decrease near to the optimal criterion, and changes in reward become difficult to judge. The gradient also depends on discriminability. For the Method of Limits it is lowest for low SNRs (see grey lines in Fig. 9D). Thus at low SNRs optimal behaviour is less likely, and decision criteria are likely to be more variable. The model would also predict that criterion for the Method of Constant Stimuli, with a reward gradient equivalent to mid-level SNRs, would have intermediate decision variability.

## Discussion

The effects of sensory and non-sensory factors in behavioural responses of ferrets in a signal-in-noise detection task were investigated by (1) applying SDT tools to analyse ferrets’ behavioural performance and (2) using a computational model to simulate behavioural trial-and-response series reproducing the experimental observations.

### The adequacy of SDT

Decision-criterion free measures of performance, *P*(c)_max_, were stable within individuals across methods. This supports the practical value of SDT in interpreting animal behaviour (Marston, 1996; Blough, 2001). It also argues against the use of methods that instead adjust for false-alarm rate (see also Long, 1994; Heffner and Heffner, 2001), which would yield different results depending upon the data collection method used. A measured ROC curve in one ferret (data not shown) further supported SDT that assumes equal variance distributions. This assumption does not always hold, especially for conditions away from threshold, in animals (Hack, 1963; Talwar and Gerstein, 1999) or in humans (see Swets, 1996; Jesteadt et al., 2003). Nevertheless, although more complex models may be required in some situations (Micheyl and Dai, 2009), both data and model suggest that a simple equal-variance SDT model offers an adequate interpretation here.

### Choice of methods in animal psychophysics

Consistent results across different methods were by no means a foregone conclusion. It is very likely that cognitive factors influence results to a greater extent than in humans, and these may depend on species, their intelligence and the naturalness of the task (Harrison, 1992). In pigeons, performance on a visual spatial acuity task was superior for the Method of Limits than the Method of Constant stimuli. The largest differences were for easier conditions (Blough, 1971) and so seem likely to be attention related. In rats performing auditory intensity discrimination, the Method of Limits gave inferior performance for ascending rather than descending stimulus differences (Terman, 1970; Terman and Terman, 1972). A likely explanation is deterioration in ‘stimulus control’ (relationship between the stimulus and the response). Instead, we only found differences for thresholds derived from adaptive tracking, consistent with observations in mice (Klink et al., 2006). However, several previous auditory studies have used tracking algorithms with success (e.g. Serafin et al., 1982; Costalupes, 1983; Shofner et al., 1993; Early et al., 2001). In rabbits, tracking has been found to be at least as stable as the Method of Constant Stimuli (Early et al., 2001; Zheng et al., 2002). In this paper, we showed that decision criterion shifted depending on stimulus statistics in the current session and recent trials. Criterion shifts are more likely in one-interval tasks used in animals, compared with two-interval 2AFC tasks more normally used in humans. Thus there is good reason to expect that non-sensory influences will be complex for adaptive tracking.

We cannot rule out that in our data a more detailed sampling of the psychometric function might have revealed differences between methods. Two-thirds of the points fall within the mid two-thirds (60–90% *P*(c)_max_) of the complete psychometric function. Greater power would be afforded by larger sampling of the mid-portion. However, the need to include a reasonable proportion of suprathreshold levels (to maintain stimulus control) limited the sampling along the mid-portion of the function.

Although the Method of Constant Stimuli and the Method of Limits gave similar results, the Method of Constant Stimuli is also an efficient way to obtain reliable estimates of detection threshold (though other methods exist which have not been tried here, Green, 1993). A bootstrap procedure employed to calculate thresholds from subgroups of trials estimated that detection thresholds stabilise at 25–40 trials per level (results not shown). Klink et al. (2006) found similarly that a reduction in the number of trials did not markedly affect thresholds using the Method of Constant Stimuli. Also, when correlating behaviour and neuronal responses (e.g. Britten et al., 1992; Atiani et al., 2009), particularly in behaving animals, the Method of Constant Stimuli is preferable as it is good practice in any physiological recordings, to randomise stimulus order.

### Criterion setting

The model was able to account for most of the variance which was not attributable to the experimental parameters (Fig. 7; Table 1). The difference between the methods was largely reproduced in the model by the assumed differences in the optimum criterion (section ‘Experimental Procedures’). Although rarely explored experimentally, a similar result was observed in pigeons, engaged in a visual task (Wixted, 1993), and in human studies (Gorea and Sagi, 2000; Agus et al., 2010). Thus it appears that in animals and humans decisions in detection employ a single decision criterion, which is quite well accounted for by mean signal distributions.

Differences in decision criterion between the methods reflected the correct strategies to maximise rewards. However, overall two additional factors were required to explain all of the data. The decision criterion for the Method of Limits was more variable at low signal levels, and of intermediate variability for the Method of Constant Stimuli. This has also been observed in humans (Maddox, 2002), and was attributed to the difficulty of refining the decision criterion when near to the optimum. Maddox (2002) also reports the last characteristic of the decision criterion we observed: more difficult trials lead to more biased responses. The more difficult a decision the more likely any natural bias is to arise. This arose naturally in the signal detection model as bias was a fixed value across different signal levels. Ferrets were trained at a high sound level on the same frequency used for testing, so have received extensive reinforcement where a high decision criterion is optimal. Furthermore, if during behaviour subjects begin with a high decision criterion and then seek to adjust it to maximise reward, then it is likely to remain higher than optimal, and more so for conditions where the gradient reward function is shallower. Thus, ferrets appear to be optimising their behaviour in a consistent way regardless of the stimulus set presented, with a strategy to maximise their rewards that is suboptimal in predictable ways.

### Sources of criterion noise

SDT does not distinguish between a broad sensory representation and variability in the decision criterion. Unsystematic variability in the criterion manifests as lower values of *d*′ and shallower psychometric functions. We might have expected sources of decision noise to affect the different collection methods differently. The Method of Constant Stimuli might be predicted to lead to a more variable criterion, if it were easier to maintain a stable criterion for a set of equally discriminable sounds of constant level. Conversely, it has been argued that presentation of a range of easy and harder stimuli helps to maintain ‘stimulus control’ in animal experiments (Blough and Blough, 1977; Klink et al., 2006). This would predict larger internal noise for the Method of Limits. However, the similarity of psychometric functions and thresholds and the fitted model parameters for the two methods argues against anything but very small differences in criterion noise between the methods.

Another potential source of decision noise was inattention. However, modelling inattention as a constant guessing rate (Wightman and Allen, 1992, chapter 4; Blough, 2001), at least, suggested inattention did not strongly influence the ferrets’ performance, with little difference between the two methods.

Ferret behavioural responses were influenced by the outcome in previous trials (Figs. 5 and 6; Table 1). Trial dependencies have been explained, using SDT, as trial-wise shifts in the decision criterion (Treisman and Williams, 1984). Unlike our data, generally in these experiments a ‘yes’ response increased the probability of a subsequent ‘yes’ response, and correction trials were not used. Rats performing frequency discrimination in a *go/no-go* task (Talwar and Gerstein, 1999), also respond more (i.e. go) following a previous response (whether a hit or false-alarm). Nevertheless, although the nature of the dependency in our data may be quite different, it was well accounted for as a shift in SDT decision criterion.

The question arises: could correction trials have induced this trial-by-trial dependency? Removal of correction trials did not change the trial dependencies, but their long term use may have led to the observed dependencies. Correction trials are widely used in animal psychophysics to instruct mistakes and to control for response bias (e.g. Blough and Blough, 1977; Kelly et al., 1986; Britten et al., 1992; Meier et al., 2011). We have tried unsuccessfully to train ferrets in the task without the use of correction trials. However, ferrets did not reach 100% correct after correction trials (Figs. 5 and 6) suggesting correction trials were not fully recognised. Rather, these responses were more strongly affected by the previous trial than correct, rewarded trials, suggesting a probabilistic dependency on previous response *and* rewards.

Simulations demonstrated that thresholds are virtually unaffected by these dependencies. Thus there may be no practical impact of correction trials on psychophysical measurements. It is, however, possible that we have underestimated their impact, if for example it is compounded by further dependencies lasting many trials. Previous models of such criterion shifts (Treisman and Williams, 1984) have considered the decaying influence of previous trials as a model parameter. Here, we did not consider such models, given that we only found significant effects two trials prior. It is also possible that both session-by-session and trial-by trial changes are both part of a more general process. The data are consistent with a standard SDT model allied to an adaptive process that adjusts the decision criterion, potentially trial-by-trial (Barraclough et al., 2004; Dayan and Niv, 2008), ensures near optimal setting of SDT decision criterion and maximises the reward gained.

## Figures and Tables

**Table 1:** Parameters used in the SDT-based model to simulate the behavioural performance obtained experimentally for each of the five ferrets tested in the signal-in-noise detection task. Also presented are (1) the mean correlation coefficient between simulated blocks of sessions and (2) the mean correlation coefficient between each experimental block of sessions and the corresponding set of simulated blocks of sessions. The correlation coefficients were calculated separately for *P*(c)_max_, hit and false alarm values (note that false alarm correlations would not be meaningful for the Method of Constant Stimuli)

Ferret	Internal s.d. (dB)	Block criterion bias (mean + s.d., dB)	Guess rate (%)	Reference level (dB SPL)	Trial shift *i* − 1	Trial shift *i* − 2	(1) sim–sim			(2) exp–sim		
							*r*^2^*P*(c)_max_	*r*^2^ hits	*r*^2^ fa	*r*^2^*P*(c)_max_	*r*^2^ hits	*r*^2^ fa
*Method of Limits*												
F1, 35	10.54	1.8 ± 1.52	1	12.54	2	1	0.93	0.87	0.84	0.90	0.82	0.74
F1, 55	11.83	1.48 ± 0.02	1	31.55	2	1	0.86	0.78	0.74	0.81	0.72	0.62
F2	9.2	3.78 ± 3.12	1	37.35	2	1.5	0.90	0.77	0.73	0.94	0.90	0.87
F3	11.29	1.03 ± 0.03	5	39.46	2.5	2	0.86	0.79	0.72	0.86	0.70	0.31
F4	11.00	1.05 ± 1.80	5	29.86	2	1.5	0.91	0.80	0.79	0.81	0.38	0.62
F5	7.24	1.85 ± 0.84	1.3	30.30	2	1	0.94	0.92	0.84	0.92	0.76	0.28
						Mean *r*^2^	0.89	0.82	0.77	0.87	0.71	0.57

							(1) sim–sim		(2) exp–sim			
							*r*^2^*P*(c)_max_	*r*^2^ hits	*r*^2^*P*(c)_max_	*r*^2^ hits		

*Method of Constant Stimuli*												
F1, 35	11	2.98 ± 0.34	1	12	2.5	1	0.92	0.95	0.80	0.88		
F1, 55	11.8	3.40 ± 0.07	1	32.76	3	2.5	0.88	0.93	0.81	0.87		
F2	8.07	3.62 ± 0.11	1	37.39	2	1.5	0.91	0.96	0.68	0.85		
F3	11.86	4.19 ± 0.06	2	40.51	3.5	3	0.90	0.95	0.90	0.86		
F4	10.50	3.20 ± 1.84	1	35.14	2	1.5	0.89	0.93	0.83	0.87		
F5	11.09	2.24 ± 0.89	2	26.29	2	1.5	0.85	0.92	0.58	0.74		
						Mean *r*^2^	0.89	0.94	0.76	0.84		
